# Supplementary material for: Expression Changes of MHC and Other Immune Genes in Frog Skin during Ontogeny
Source: Animals (Basel). 2020 Jan 6;10(1):91. doi: 10.3390/ani10010091 (PMC7022564; doi:10.3390/ani10010091)
Supplement: Supplementary file 1 [file animals-10-00091-s001.pdf]

Supplementary Methods

Preliminary PCR of MHC class I and II in Xenopus frogs

To support our findings following transcriptome analyses, we conducted preliminary PCR in *Xenopus* tadpoles to check for general expression patterns based on presence of MHC amplicons of the correct size visualized after gel electrophoresis. For template cDNA, we utilized the same *X.tropicalis* cDNA samples used for transcriptome analyses (three stages, three replicates each) as well as the equivalent cDNA samples from *X. laevis* (same rearing institute, stages, number of replicates, and extraction methods). Although the two *Xenopus* species have morphological similarities, there may be genetic differences since they differ in ploidy and have an estimated divergence time of 48 MYA (Session et al 2016). We used the same traditional PCR amplification procedures described in the main text, but with approximately 100 ng template cDNA and primers (see Table below) designed from conserved regions of *X. tropicalis* and *X. laevis* sequence alignments (GenBank accessions- *MHC class I*: AF185582, AF185580, NM_001142006, AY204558; *MHC class II*: NM_001045794, NM_001135067, BC092157, D13687.1). We expect only a single locus of each MHC class I to be amplified as there is only one detectable classical class I locus in *Xenopus* (Flajnik et al 1999). Meanwhile, class II primers were designed based on alleles from different loci, thus multiple MHC class II loci may be amplified. Representative amplicons (one individual per stage per sequence when amplicon was visible) were also sequenced to confirm that amplified sequences represented either MHC class I (763 bp), MHC class II (693 bp), or reference gene GAPDH (639 bp).

Summary of primers used for traditional PCR in Xenopus.

**Target gene**	**Target species**	**Boundary crossed**	**Forward primer (5′ – 3′)**	**Reverse primer (5′ – 3′)**	**Amplicon length (bp)**
MHC class I	*Xenopus*	exon 2 - 4	CAGTCAYTCCCTGCGCTAYT	CTGTCACCYTCMYTGGGTGT	763
MHC class II beta DBB	*Xenopus*	exon 1 - 4	GKGGRRTATCAGTGCGAGTT	AGATGGATCCMAGCACAAAG	693
GAPDH	*Xenopus*	exon 3 - 6	TTAAGTGGGGMGATGCTGGT	CRGCATCAAAGATGGAGGAR	639

References:

Flajnik, M. F. et al. Two ancient allelic lineages at the single classical class I locus in the Xenopus MHC. J. Immunol. 163, 3826–3833 (1999).

Session, A. M. et al. Genome evolution in the allotetraploid frog Xenopus laevis. Nature 538, 336–343 (2016).

Supplementary Figures

Figure S1. Gel electrophoresis following PCR amplification of GAPDH, MHC class I, and MHC class II from early-, mid-, and late-larval stages of *Xenopus laevis* and *X. tropicalis* tadpole skin cDNA samples (n =3 per stage per species).

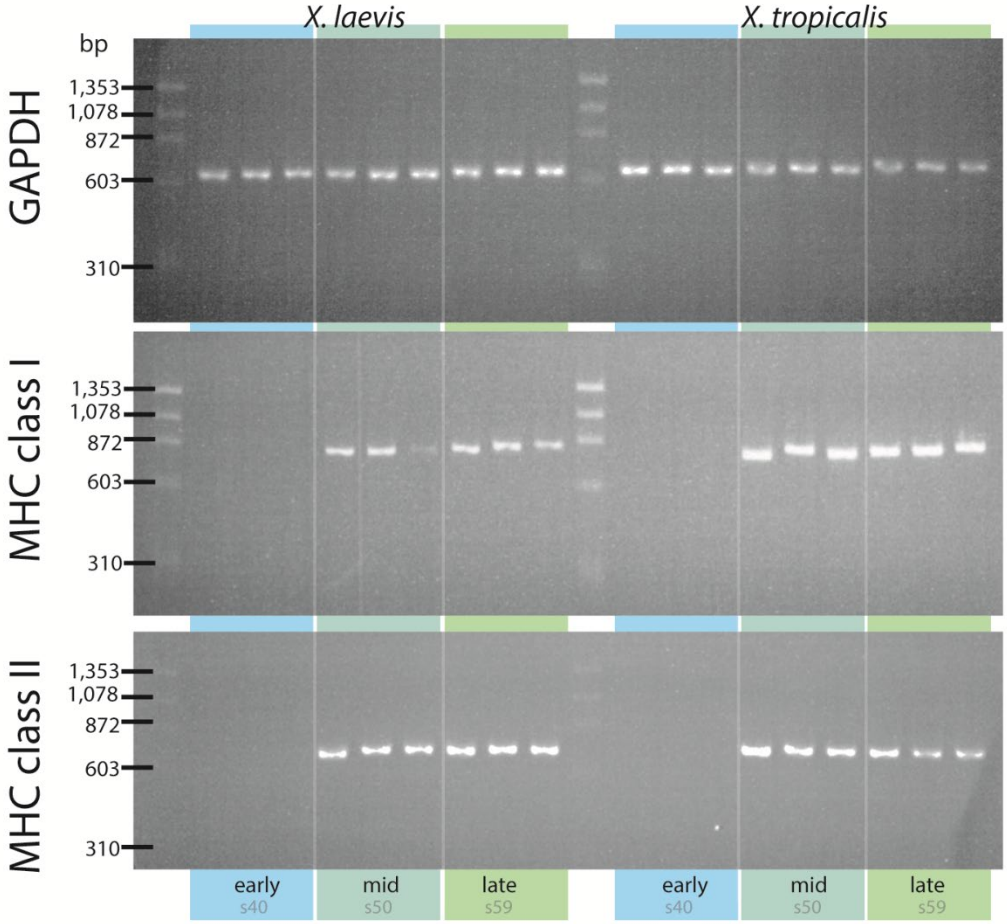

Supplementary Tables

Table S1. Approximation of tadpole stages by Gosner (1960) to those of Tahara (1959, 1974), Niewkoop and Faber (1994, N/F), Shumway (1940) and Taylor and Kollros (1946, T/K). Table adapted from McDiarmid and Altig (1999) with additional approximations from Tahara (1958, 1974). *Rana japonica* stages 33 - 35 and 39 did not have equivalent stages matched to that of *Rana pipiens* and thus were approximated (in italics). Stages used for our qPCR study in *R. ornativentris* (which is closely related to *R. japonica* are in bold. 'Early-', 'mid-', and 'late-'larval stages used for cDNA library construction and transcriptome analyses in both *R. ornativentris* and *X. tropicalis* (closely related to *X. laevis*) are indicated by boxes.

**Tahara** *Rana japonica*	**Gosner**	**N/F** *Xenopus laevis*	**Shumway** *Rana pipiens*	**T/K** *Rana pipiens*
1	1	1	-	-
2	2	1*	2	-
3	3	2	3	-
4	4	3	4	-
5	5	4	5	-
6	6	5	6	-
7	7	6	7	-
8	8	7	8	-
9	9	9	9	-
10a	10	10	10	-
11	11	11	11	-
12	12	12	12	-
13a	13	13	13	-
14	14	14	14	-
15	15	17	15	-
16	16	19	16	-
17	17	23	17	-
18	18	26	18	-
19	19	30	19	-
**20a**	20	32	20	-
21	21	35	21	-
-	-	**40 'early'**	-	-
22	22	41	22	-
23	23	43	23	-
**24 'early'**	24	44	24	-
**25**	25	46	25	-
-	26	48	-	I
26	27	49	-	II
27	28	**50 'mid'**	-	III
**28 'mid'**	29	51	-	IV
**29**	30	52	-	V
-	31	53	-	VI
-	32	53*	-	VII
30	33	53**	-	VIII
31	34	54	-	IX
32	35	54*	-	X
33	36	54**	-	XI
34	37	55	-	XII
-	38	56*	-	XIII
35	39	56	-	XIV
-	40	56*	-	XV-XVII
36	41	58	-	XVIII-XIX
**37**	42	**59 'late'**	-	XX
-	43	61	-	XXI
38	44	62	-	XXII
39	45	63	-	XXIII-XXIV
**40 'late'**	46	66	-	XXV

Table S2. Normalized (and log-normalized) MHC class I and II expression values (average ± S.D.) of *Rana ornativentris* tadpole skin samples and other tissues.

Sample	Normalized MHC-I	Log-normalized MHC-I	Normalized MHC-II	Log-normalized MHC-II
stage 20	0.0042 ± 0.0038	-5.88 ± 0.98	0.0001 ± 0.0001	-9.36 ± 0.83
stage 24/25	0.0026 ± 0.0016	-6.11 ± 0.65	0.0001 ± 0.0002	-9.17 ± 0.76
stage 28/29	0.1192 ± 0.1032	-2.42 ± 0.78	0.0115 ± 0.0127	-4.93 ± 0.98
stage 33	0.1496 ± 0.0859	-2.11 ± 0.76	0.0126 ± 0.0090	-4.63 ± 0.83
stage 37	0.2309 ± 0.1647	-1.85 ± 1.15	0.0272 ± 0.0188	-3.87 ± 0.82
stage 40	0.2052 ± 0.1253	-1.73 ± 0.57	0.0191 ± 0.0129	-4.14 ± 0.62
stage 24/25 body	0.0050 ± 0.0033	-5.50 ± 0.72	0.0004 ± 0.0002	-7.89 ± 0.46
Adult skin	0.0868 ± 0.0423	-2.67 ± 0.90	0.0198 ± 0.0309	-4.67 ± 1.27
Adult spleen	0.2786 ± 0.2382	-1.64 ± 1.00	0.1278 ± 0.1182	-2.45 ± 1.04

Table S3. Number of differentially expressed transcripts in pairwise comparisons of samples in *Rana ornativentris* (Ro) or *Xenopus tropicalis* (Xt). The values in parentheses represent the number of Gene Ontology (GO) terms within parent term 'Biological processes' (BP) that are significantly enriched in each sample (count ≥ 3, p < 0.10); the full list of immune-related enriched GO terms are summarized in Table S5 and S6. E-early, M-mid, and L-late larval stages.

Pairwise comparison		*No.of differentially expressed transcripts (GO BP terms)*
**Ro skin E x M**	E up	1471 (190)
	M up	1100 (165)
Ro skin E x L	E up	2008 (273)
	L up	741 (37)
Ro skin M x L	M up	1673 (233)
	L up	794 (61)
Ro E skin x bod	Skin up	10 (0)
	Bod up	57 (24)
Ro M skin x bod	Skin up	225 (21)
	Bod up	1133 (277)
Ro E/M skin x bod	Skin up	17 (0)
	Bod up	366 (59)
Xt skin *E x M*	E up	1309 (289)
	M up	2207 (183)
Xt skin E x L	E up	3956 (434)
	L up	3001 (166)
Xt skin M x L	M up	197 (89)
	L up	136 (27)

Table S4. Top 10 enriched GO terms in each pairwise comparison of *Rana ornativentris* or *Xenopus tropicalis* tadpole samples. No GO terms were enriched in skin over body samples at E and E+M stages. E-early, M-mid, and L-late larval stages.

GO term	Count	P-value
R.ornativentris skin E > M		
nervous system development	111	4.30E-09
neuron projection development	43	2.00E-06
single-organism process	625	4.50E-06
neuron differentiation	60	2.30E-05
neuron development	49	3.10E-05
generation of neurons	63	3.50E-05
single-multicellular organism process	214	4.10E-05
neuron projection morphogenesis	33	4.20E-05
neurogenesis	68	4.30E-05
cell differentiation	122	4.60E-05
R.ornativentris skin M > E		
ribonucleoprotein complex biogenesis	143	2.80E-30
ribosome biogenesis	110	1.20E-27
organonitrogen compound metabolic process	353	8.30E-27
protein metabolic process	584	9.80E-26
peptide metabolic process	192	1.10E-25
translation	170	1.70E-25
peptide biosynthetic process	171	3.00E-25
cellular protein metabolic process	544	2.70E-24
amide biosynthetic process	181	7.20E-24
cellular amide metabolic process	210	3.00E-22
R.ornativentris skin E > L		
myofibril assembly	10	1.10E-04
skeletal muscle contraction	6	2.20E-04
nervous system development	20	2.90E-04
skeletal muscle cell differentiation	5	1.40E-03
homophilic cell adhesion via plasma membrane adhesion molecules	21	1.50E-03
regulation of transcription, DNA-templated	84	1.60E-03
muscle contraction	7	2.50E-03
sodium ion transport	8	2.80E-03
synaptic transmission, glycinergic	5	3.80E-03
regulation of axon extension	5	3.80E-03
R.ornativentris skin L > E		
arachidonic acid metabolic process	10	7.10E-04
antigen processing and presentation	9	9.20E-04
immune response	26	1.30E-03
long-chain fatty acid metabolic process	10	1.30E-03
epoxygenase P450 pathway	9	1.40E-03
icosanoid metabolic process	10	2.20E-03
unsaturated fatty acid metabolic process	10	4.20E-03
regulation of autophagy	8	6.30E-03
programmed cell death	34	9.90E-03
immune system process	41	1.30E-02
R.ornativentris skin M > L		
ribonucleoprotein complex biogenesis	141	9.10E-26
ribosome biogenesis	109	2.60E-24
organonitrogen compound metabolic process	358	1.10E-22
translation	171	1.60E-22
peptide biosynthetic process	172	2.90E-22
peptide metabolic process	192	6.20E-22
cellular protein metabolic process	564	8.20E-22
protein metabolic process	599	1.90E-21
amide biosynthetic process	183	3.00E-21
organonitrogen compound biosynthetic process	265	1.70E-20
R.ornativentris skin L > M		
regulation of autophagy	9	2.50E-03

protein import into nucleus	10	1.30E-02
single-organism nuclear import	10	1.30E-02
nuclear import	10	1.30E-02
protein targeting to nucleus	10	1.30E-02
protein localization to nucleus	10	1.40E-02
transmembrane receptor protein tyrosine kinase signaling pathway	20	1.70E-02
cytoskeleton organization	39	2.40E-02
cell surface receptor signaling pathway	66	2.60E-02
positive regulation of gene expression	28	2.80E-02
R.ornativentris E body > skin		
visual perception	5	1.00E-05
sensory perception of light stimulus	5	1.10E-05
sensory perception	7	1.30E-05
neurological system process	7	2.30E-05
system process	7	4.60E-04
multicellular organismal process	13	3.50E-03
response to light stimulus	3	1.10E-02
response to radiation	3	2.30E-02
cellular response to stimulus	12	3.40E-02
G-protein coupled receptor signaling pathway	4	7.00E-01
R.ornativentris M skin > body		
regulation of extent of cell growth	5	3.30E-04
embryonic eye morphogenesis	4	3.80E-04
regulation of cell growth	6	4.30E-04
regulation of axonogenesis	5	7.90E-04
regulation of developmental growth	5	8.60E-04
regulation of growth	6	1.10E-03
regulation of cell size	5	1.60E-03
regulation of cell morphogenesis involved in differentiation	5	1.70E-03
regulation of anatomical structure morphogenesis	8	2.00E-03
regulation of neuron projection development	5	2.60E-03
R.ornativentris M body > skin		
organonitrogen compound metabolic process	131	3.20E-15
amide biosynthetic process	75	3.40E-15
peptide biosynthetic process	70	6.20E-15
translation	69	1.10E-14
peptide metabolic process	74	1.90E-13
cellular amide metabolic process	82	4.10E-13
organonitrogen compound biosynthetic process	94	1.80E-11
small molecule metabolic process	96	7.30E-09
organic acid metabolic process	58	1.30E-07
oxoacid metabolic process	52	1.10E-06
R.ornativentris M+E body > skin		
blood coagulation	10	4.50E-08
hemostasis	10	4.50E-08
coagulation	10	7.50E-08
regulation of body fluid levels	10	2.30E-07
wound healing	12	6.20E-07
response to wounding	12	2.10E-06
protein activation cascade	6	2.60E-06
visual perception	9	9.00E-06
sensory perception of light stimulus	9	1.00E-05
regulation of hemostasis	4	6.80E-04
Xenopus tropicalis skin E >M		
visual perception	23	3.60E-14
sensory perception of light stimulus	23	5.90E-14
organic cyclic compound biosynthetic process	139	1.40E-07
multicellular organismal process	174	1.70E-07
organic cyclic compound metabolic process	196	5.90E-07
aromatic compound biosynthetic process	131	2.50E-06
heterocycle biosynthetic process	130	4.30E-06
nucleobase-containing compound biosynthetic process	126	8.30E-06
digestive system development	15	1.30E-05

cellular aromatic compound metabolic process	185	1.90E-05
Xenopus tropicalis skin M > E		
immune response	49	1.60E-15
defense response	46	2.50E-15
immune system process	75	6.50E-15
innate immune response	29	2.60E-12
inflammatory response	26	2.30E-11
response to biotic stimulus	27	1.10E-08
regulation of immune system process	27	1.70E-08
response to other organism	25	3.40E-08
response to external biotic stimulus	25	3.40E-08
regulation of immune response	20	5.20E-08
Xenopus tropicalis skin E> L		
nervous system development	211	9.20E-18
microtubule-based process	92	5.10E-12
neurogenesis	135	5.80E-11
microtubule cytoskeleton organization	58	1.10E-10
single-multicellular organism process	418	1.20E-10
system development	331	1.70E-10
generation of neurons	121	8.30E-10
neuron differentiation	113	1.70E-09
multicellular organismal process	468	2.30E-09
cell cycle process	96	2.40E-09
Xenopus tropicalis skin L > E		
defense response	55	9.80E-16
immune response	57	8.30E-15
inflammatory response	34	1.30E-14
immune system process	89	1.20E-13
innate immune response	29	5.40E-09
regulation of immune system process	31	6.90E-08
regulation of Ras protein signal transduction	26	3.30E-07
response to biotic stimulus	29	5.90E-07
actomyosin structure organization	18	8.70E-07
response to external biotic stimulus	27	1.10E-06
Xenopus tropicalis skin M > L		
microtubule-based process	14	5.70E-03
cell cycle	16	1.80E-02
DNA-dependent DNA replication	7	1.60E-02
single-organism cellular process	79	1.30E-02
regulation of DNA-dependent DNA replication	4	2.70E-02
single-organism process	86	2.30E-02
DNA replication initiation	5	2.10E-02
microtubule-based movement	8	1.90E-02
regulation of DNA replication	4	4.90E-02
cell cycle process	12	5.60E-02
Xenopus tropicalis skin L > M		
muscle structure development	8	1.40E-05
striated muscle cell development	6	3.70E-05
muscle cell development	6	5.40E-05
striated muscle cell differentiation	6	1.20E-04
muscle cell differentiation	6	2.60E-04
myofibril assembly	4	8.00E-04
actomyosin structure organization	4	3.00E-03
cardiocyte differentiation	3	9.50E-03
cell development	9	1.80E-02
organelle organization	15	2.40E-02

Table S5. Immune-related GO terms enriched in specific stages or tissues of *Rana ornativentris* tadpoles. No immune-related GO terms were significantly enriched in early stage tadpoles. E-early, M-mid, and L-late larval stages.

Immune-related GO term	Count	P-value
Skin M (s28) > skin E (s24/25)		
GO:0019882~antigen processing and presentation	15	1.30E-02
GO:0002483 ~antigen processing and presentation of endogenous peptide antigen	6	2.90E-02
GO:0019885antigen processing and presentation of endogenous peptide antigen via MHC class I	6	2.90E-02
GO:0002474~antigen processing and presentation of peptide antigen via MHC class I	6	2.90E-02
GO:0048002~antigen processing and presentation of peptide antigen	6	2.90E-02
GO:0019883~antigen processing and presentation of endogenous antigen	6	2.90E-02
GO:0002520~immune system development	55	6.80E-02
GO:0002485~antigen processing and presentation of endogenous peptide antigen via MHC class I via ER pathway, TAP-dependent	5	7.40E-02
GO:0002585~positive regulation of antigen processing and presentation of peptide antigen	5	7.40E-02
GO:0002478 ~antigen processing and presentation of exogenous peptide antigen	5	7.40E-02
GO:0002589 ~regulation of antigen processing and presentation of peptide antigen via MHC class I	5	7.40E-02
GO:0002481~antigen processing and presentation of exogenous protein antigen via MHC class Ib, TAP-dependent	5	7.40E-02
GO:0019884 ~antigen processing and presentation of exogenous antigen	5	7.40E-02
GO:0002579 ~positive regulation of antigen processing and presentation	5	7.40E-02
GO:0002488 ~antigen processing and presentation of endogenous peptide antigen via MHC class Ib via ER pathway	5	7.40E-02
GO:0002583 ~regulation of antigen processing and presentation of peptide antigen	5	7.40E-02
GO:0002428~antigen processing and presentation of peptide antigen via MHC class Ib	5	7.40E-02
GO:0002591~positive regulation of antigen processing and presentation of peptide antigen via MHC class I	5	7.40E-02
GO:0002475 ~antigen processing and presentation via MHC class Ib	5	7.40E-02
GO:0002489~antigen processing and presentation of endogenous peptide antigen via MHC class Ib via ER pathway, TAP-dependent	5	7.40E-02
GO:0002477 ~antigen processing and presentation of exogenous peptide antigen via MHC class Ib	5	7.40E-02
GO:0002577 ~regulation of antigen processing and presentation	5	7.40E-02
GO:0002484~antigen processing and presentation of endogenous peptide antigen via MHC class I via ER pathway	5	7.40E-02
GO:0002476~antigen processing and presentation of endogenous peptide antigen via MHC class Ib	5	7.40E-02
Skin L (s40) > skin E (s24/25)		
GO:0019882~antigen processing and presentation	9	9.20E-04
GO:0006955~immune response	26	1.30E-03
GO:0002376~immune system process	41	1.30E-02
GO:0045087~innate immune response	12	3.60E-02
Skin L (s40) > skin M (s28)		
GO:0002526~acute inflammatory response	3	3.00E-02
Body E/M > skin E/M		
GO:0006955~immune response	11	6.30E-03

Table S6. Immune-related GO terms enriched in skin of mid and/or late (M/L) compared to early (E) larval stages of *Xenopus tropicalis* tadpoles. No immune-related GO terms were significantly enriched in the early-larval stage, as well as in the comparison between mid- and late-larval stage tadpoles.

Immune-related GO term (*M/L > E*)	Count (M_up/ L_up)	P-value (M_up/ L_up)
GO:0006955~immune response	49 / 57	1.60E-15/ 8.30E-15
GO:0002376~immune system process	75 / 89	6.50E-15/ 1.20E-13
GO:0006952~defense response	46 / 55	2.50E-15/ 9.80E-16
GO:0045087~innate immune response	29 / 29	2.60E-12/ 5.40E-09
GO:0050776~regulation of immune response	20 / 21	5.20E-08/ 1.90E-06
GO:0006954~inflammatory response	26 / 34	2.30E-11/ 1.30E-14
GO:0002682~regulation of immune system process	27/31	1.70E-08/ 6.90E-08
GO:0002684~positive regulation of immune system process	20/21	3.90E-07/ 1.30E-05
GO:0002253~activation of immune response	15/ 16	1.30E-06/ 1.20E-05
GO:0050778~positive regulation of immune response	16/ 16	2.10E-06/ 1.10E-04
GO:0002764~immune response-regulating signaling pathway	14/15	2.50E-06/ 1.70E-05
GO:0019882~antigen processing and presentation	12/12	4.30E-06/ 9.40E-05
GO:0002757~immune response-activating signal transduction	13/14	1.10E-05/ 6.30E-05
GO:0045089~positive regulation of innate immune response	10/9	1.90E-05/ 1.40E-03
GO:0031349~positive regulation of defense response	10/9	1.90E-05/ 1.40E-03
GO:0002758~innate immune response-activating signal transduction	9/9	2.10E-05/ 2.20E-04
GO:0002221~pattern recognition receptor signaling pathway	9/9	2.10E-05/ 2.20E-04
GO:0002224~toll-like receptor signaling pathway	9/9	2.10E-05/ 2.20E-04
GO:0002218~activation of innate immune response	9/9	3.30E-05/ 3.40E-04
GO:0009617~response to bacterium	14/16	5.40E-05/ 1.10E-04
GO:0034097~response to cytokine	15/16	1.10E-04/ 9.70E-04
GO:0045088~regulation of innate immune response	10/10	1.10E-04/ 1.20E-03
GO:0002755~MyD88-dependent toll-like receptor signaling pathway	6/7	1.70E-04/ 5.40E-05
GO:0009615~response to virus	9/9	3.70E-04/ 3.20E-03
GO:0002252~immune effector process	12/11	3.80E-04/ 1.50E-02
GO:0019221~cytokine-mediated signaling pathway	11/10	1.50E-03/ 3.90E-02
GO:0002237~response to molecule of bacterial origin	7/10	2.50E-03/ 7.40E-05
GO:0071345~cellular response to cytokine stimulus	11/11	3.30E-03/ 3.00E-02
GO:0051607~defense response to virus	6/6	1.30E-02/ 4.70E-02
GO:0002526~acute inflammatory response	3/3	2.70E-02/ 5.00E-02
GO:0050727 regulation of inflammatory response	4/4	2.80E-02/ 6.50E-02
GO:0043300 regulation of leukocyte degranulation	3/-	4.30E-02/ -
GO:0043299~leukocyte degranulation	3/ -	4.30E-02/ -
GO:0002279~mast cell activation involved in immune response	3/ -	4.30E-02/ -
GO:0002886~regulation of myeloid leukocyte mediated immunity	3/ -	4.30E-02/ -
GO:0033006~regulation of mast cell activation involved in immune response	3/ -	4.30E-02/ -
GO:0002768~immune response-regulating cell surface receptor signaling pathway	5/ -	4.70E-02/ -
GO:0001816~cytokine production	4/ -	5.80E-02/ -
GO:0001819~positive regulation of cytokine production	3/ -	6.10E-02/ -
GO:0002275~myeloid cell activation involved in immune response	3/ -	6.10E-02/ -
GO:0042742~defense response to bacterium	5/ -	6.40E-02/ -
GO:0001817~regulation of cytokine production	4/ -	7.00E-02/ -
GO:0002444~myeloid leukocyte mediated immunity	3/ -	8.10E-02/ -
GO:0002444~regulation of leukocyte mediated immunity	3/ -	8.10E-02/ -
GO:0050900~leukocyte migration	5/8	8.30E-02/ 6.30E-03
GO:0050851~antigen receptor-mediated signaling pathway	4/ -	9.70E-02/ -
GO:0002250~adaptive immune response	4/ -	9.70E-02/ -
GO:0019723~leukocyte mediated immunity	4/ -	9.70E-02/ -
GO:0071593~lymphocyte aggregation	-/ 5	-/ 7.60E-02
GO:0070486~leukocyte aggregation	-/ 5	-/ 7.60E-02
GO:0070489~T cell aggregation	-/ 5	-/ 7.60E-02
GO:0071610~T cell activation	-/ 5	-/ 7.60E-02
GO:0046649~lymphocyte activation	-/ 8	-/ 8.80E-02
